# Phagocytic hemocytes, independent of pericardial cells, modulate cellular immune responses on the dorsal vessel of mosquitoes, including the infection-induced reduction of the heart rate

**DOI:** 10.1007/s00441-025-04011-y

**Published:** 2025-09-19

**Authors:** Cole J. Meier, Shabbir Ahmed, Tania Y. Estévez-Lao, Julián F. Hillyer

**Affiliations:** Department of Biological Sciences, Vanderbilt University, VU Station B 35-16342, Nashville, TN 37235 USA

**Keywords:** Heart, Hemocyte, Mosquito, Nitric oxide, Phagocytosis

## Abstract

Infection induces the aggregation of hemocytes on the dorsal vessel of mosquitoes. These hemocytes, called periostial hemocytes, phagocytose pathogens and produce immune factors on the abdominal portion of the dorsal vessel, called the heart. One of these immune factors, nitric oxide, is a pleiotropic free radical that is an antimicrobial and a heartbeat reducer. But nitric oxide is not just produced by hemocytes. It is also synthesized by pericardial cells that flank the heart, and other tissues. To determine whether it is the periostial hemocytes or the pericardial cells that modulate the heart following infection, we chemically ablated the hemocytes using clodronate liposomes and measured immune responses and heart physiology. We demonstrate that clodronate liposomes ablate the sessile hemocytes, including the periostial hemocytes, while leaving the pericardial cells and heart integrity unaffected. Moreover, ablating hemocytes abolishes the phagocytosis of bacteria, alters the deposition of melanized bacteria, and decreases nitric oxide synthase activity on the heart. Importantly, hemocyte ablation eliminates the infection induced reduction of the heart rate, mainly by modifying the anterograde heart rate. Therefore, periostial hemocytes drive immune responses on the heart and infection-induced changes to circulatory physiology.

**Supplementary Information:**

The online version contains supplementary material available at 10.1007/s00441-025-04011-y.

## Introduction

Hemocytes are the primary immune cells of insects (Eleftherianos, et al. 2021; Hillyer 2016; Strand 2008). They phagocytose, encapsulate and nodulate pathogens that have entered the hemocoel, secrete humoral factors with lytic activity, and produce the enzymes that kill pathogens by encasing them in a dark proteinaceous capsule via a cascade called melanization (Hillyer, et al. 2003a, 2003b; Lavine, et al. 2005; Pech and Strand 1995; Ratcliffe and Gagen 1977). But hemocytes are also involved in non-immune processes (Stanley, et al. 2023). They are critical for wound healing, egg chorion tanning, molting, tissue repair, clearing apoptotic cells, and lipoprotein synthesis (Babcock, et al. 2008; Kiger, et al. 2001; Kim, et al. 2004, 2005; King and Hillyer 2013; Lai, et al. 2001; Manaka, et al. 2004; Whitehead 2011). Another non-immune function of hemocytes is the modulation of the circulatory system, which occurs when hemocytes produce nitric oxide while on the surface of the heart (da Silva, et al. 2012; Estevez-Lao, et al. 2025, 2020). 

The primary circulatory organ in insects is the dorsal vessel, which is divided into an aorta in the thorax and a heart in the abdomen (Hillyer and Pass 2020). The aorta periodically contracts, but it is mainly a conduit for hemolymph being propelled by the heart (Markou and Theophilidis 2000; Sigle and Hillyer 2018b). The heart is composed of helically coiled muscle that rhythmically contracts in a wave-like manner (Ejaz and Lange 2008; Glenn, et al. 2010). In adult holometabolous insects, the heart periodically alternates between contracting in the anterograde (toward the head) and retrograde (toward the posterior of the abdomen) directions (Gerould 1933). When contracting anterograde, hemolymph enters the heart via ostia (valves) in the anterior of each abdominal segment and exits via an excurrent opening in the anterior of the aorta (Glenn, et al. 2010; Sigle and Hillyer 2018b). When contracting retrograde, hemolymph enters the heart via ostia located at the thoraco-abdominal junction and exits via an excurrent opening in the posterior of the heart. The resulting circulation of hemolymph transports nutrients, hormones and waste, maintains homeostasis, facilitates gas exchange via tracheal ventilation, disseminates immune factors and cells, and thermoregulates (Hillyer and Pass 2020).

The immune and circulatory systems of insects, and perhaps Pancrustacea in general, are functionally integrated (Hillyer 2015; Yan and Hillyer 2020). In insects, sessile hemocytes, called periostial hemocytes, reside around the heart’s ostia, where they internalize pathogens via phagocytosis and enzymatically degrade them, and secrete humoral effectors in regions of high hemolymph flow (King and Hillyer 2012; Sigle and Hillyer 2016). An infection induces the migration of additional hemocytes to the periostial regions of the heart, where they amplify the immune response. Periostial hemocytes produce nitric oxide and lysozymes, which have antimicrobial and cardiomodulatory activity (da Silva, et al. 2012; Estevez-Lao, et al. 2020).

In mosquitoes, an infection reduces the heart rate, and this is mediated by nitric oxide and lysozymes that are produced on the heart (Estevez-Lao, et al. 2020). Because, on the heart, periostial hemocytes are the largest producers of nitric oxide and the enzyme that produces it, called nitric oxide synthase, it stands to reason that these are the cells that modulate heart physiology during an infection. However, it is possible that pericardial cells also contribute to this phenotype. Pericardial cells flank the heart along its entire length (Barbosa da Silva et al. 2019; King and Hillyer 2012; League and Hillyer 2016), and these cells produce antimicrobial molecules like cecropin, lysozymes and nitric oxide (Bergmann, et al. 2024; Cardoso-Jaime, et al. 2021, 2023; Hernandez-Martinez, et al. 2013).

To determine whether it is the periostial hemocytes or the pericardial cells that modulate circulatory physiology following infection, we chemically ablated the hemocytes using clodronate liposomes (Kwon and Smith 2019; Ramesh Kumar, et al. 2021). We demonstrate that clodronate liposomes ablate sessile hemocytes without impacting the pericardial cells or the integrity of the heart. Moreover, ablating hemocytes abolishes phagocytic responses on the heart, decreases nitric oxide synthase activity, and eliminates the infection induced reduction in the heart rate. Altogether, this demonstrates that periostial hemocytes, independent of pericardial cells, drive immune responses on the surface of the dorsal vessel and infection-induced changes to circulatory physiology.

## Materials and methods

### Mosquito rearing

*Anopheles gambiae* Giles sensu stricto (G3 strain; Diptera: Culicidae) were reared on a 12 h:12 h light:dark photoperiod at 27 °C and a relative humidity of 75% (Meier, et al. 2023). Eggs were hatched in deionized water and larvae were fed a koi food and yeast mixture. Adults were fed 10% sucrose ad libitum, and experiments were initiated on 2-day-old adult female mosquitoes. Only females were used because this is the only sex that blood feeds and transmits disease (Becker, et al. 2020).

### Liposome treatment, bacterial infection, hemocyte staining, pericardial cell staining, muscle staining, and abdomen collection

Liposome treatment was done using the Standard Macrophage Depletion Kit (Clodrosome + Encapsome; Encapsula NanoSciences, Brentwood, TN) as described (Kwon and Smith 2019, 2025; Ramesh Kumar, et al. 2021). Encapsome (LP) is an empty liposome (a liposome control) whereas Clodrosome (CLD) contains encapsulated clodronate. LP and CLD were diluted 1:5 in phosphate-buffered saline (PBS, pH 7.2). Then, 69 nL of either PBS (buffer control) or a liposome solution (either LP or CLD in PBS) was injected into the thoracic anepisternal cleft using a Nanoject III (Drummond Scientific Company, Broomall, PA, USA).

For infections, GFP-expressing, tetracycline resistant *Escherichia coli* (modified DH5-alpha) were grown overnight at 37 °C in Luria–Bertani broth (LB). Cultures were diluted to OD_600_ of 2, and 69 nL was injected into the thoracic anepisternal cleft using the Nanoject III. This represents an approximate dose of 22,000 bacteria per mosquito.

For hemocyte staining, live mosquitoes were injected with 200 nL of 67 µM Vybrant CM-DiI Cell-Labeling Solution (Invitrogen, Carlsbad, California) and 0.54 mM Hoechst 33342 (Invitrogen) in PBS to label the hemocytes and cell nuclei, respectively, and incubated at room temperature for 10 min (King and Hillyer 2012; Meier, et al. 2024). CM-DiI is a lipophilic dye that has a high affinity for membranes. In live (unfixed) mosquitoes, CM-DiI injected into the hemocoel does not bind the plasma membrane of cells that are surrounded by a basal lamina, which is most tissues. Instead, it binds the plasma membrane of hemocytes and is then phagocytosed (King and Hillyer 2012). Hoechst 33342 is a permeable dye that fluoresces when bound to the DNA of any cell; it labels the nuclei of all cells (King and Hillyer 2012, 2013). For pericardial cell staining, live mosquitoes were injected 200 nL of 0.2 mg/mL Alexa Fluor conjugated goat anti-rabbit IgG (H + L; either 488 or 568 nm; Invitrogen) in PBS and incubated for 1 h (King and Hillyer 2012). Pericardial cells, also known as nephrocytes, filter the hemolymph to maintain homeostasis (Troha, et al. 2019). Therefore, injected foreign antibodies are filtered from the hemolymph by the pericardial cells, and in doing so, the dye that is conjugated to the antibodies makes these cells fluorescent (King and Hillyer 2012). For muscle staining, live mosquitoes were intrathoracically injected with 200 nL of 0.1% Triton X-100 (Thermo Fisher Scientific Inc., Waltham, MA, USA), 0.3 μmol/L phalloidin-Alexa Fluor 488 (Invitrogen) and 16% paraformaldehyde (Electron Microscopy Sciences, Hatfield, PA) in PBS and incubated for 15 min (Glenn, et al. 2010). Labeling combinations were done as described (King and Hillyer 2012).

For abdomen collection, following staining the mosquitoes were injected with 200 nL of 4% paraformaldehyde. After 10 min, each abdomen was separated from the thorax, and the abdomen was bisected along the coronal plane to separate the dorsal and ventral sides. The dorsal abdomen was transferred to PBS containing 0.1% Triton X-100, the organs not attached to the tergum were removed, and the dorsal abdomen was mounted on a glass slide with Vectashield Antifade Mounting Medium (Vector Laboratories, Newark, CA).

### Imaging of hemocytes, pericardial cells and muscle, and hemocyte quantification

Z-stacks of the dorsal abdomen were acquired at 5 µm increments using epifluorescence and bright field trans-illumination on an Eclipse Ni-E compound microscope (Nikon Corporation, Tokyo, Japan) as described (Meier, et al. 2024). Images were captured using a Nikon Digital Sight DS-Qi1 monochrome digital camera or a pco.panda 4.2 sCMOS camera (Excelitas Technologies Corp., Pittsburgh, PA) connected to Nikon’s Advanced Research NIS Elements software.

A cell was defined as a hemocyte if it labeled with both CM-DiI (red fluorescence) and Hoechst 33342 (blue fluorescence), and measured ~ 10–20 µm in diameter (King and Hillyer 2012; Sigle and Hillyer 2016). Hemocytes were considered periostial if residing immediately next to an ostium in abdominal segments 2–7, or contiguous with a cluster of hemocytes residing next to an ostium. In naïve mosquitoes, mosquitoes were treated with liposomes for 24 or 48 h and the hemocytes were then visualized. In infected mosquitoes, mosquitoes were treated with liposomes for 24 h, then infected for 24 h, and the hemocytes were then visualized. For each experiment, three independent trials were conducted. A total of 14–24 mosquitoes were assayed per treatment group. Only uninfected (naïve) and infected mosquitoes were assayed; injured mosquitoes were not quantified because injury does not induce periostial hemocyte aggregation (King and Hillyer 2012; Sigle and Hillyer 2016).

Pericardial cells were identified by their size and position along the heart (Barbosa da Silva et al. 2019; Cardoso-Jaime, et al. 2021; King and Hillyer 2012; League and Hillyer 2016). Muscle was identified by its helical arrangement along the abdomen (Andereck, et al. 2010; Glenn, et al. 2010). For qualitative analysis of pericardial cells and muscle, 8–10 abdomens were analyzed per treatment group.

### cDNA synthesis and quantitative real-time PCR (qPCR)

For each treatment, the dorsal abdomens of 20 mosquitoes were isolated and pooled for cDNA synthesis (Meier, et al. 2024). The dorsal abdomens were homogenized using a mechanical pestle, and the RNA was extracted in TRIzol Reagent (Invitrogen). The RNA was repurified with the RNeasy Mini Kit (Qiagen, Valencia, CA, USA), and up to 5 μg of RNA was used to synthesize cDNA using an Oligo (dT)_20_ primer and the SuperScript III First-Strand Synthesis System for RT-PCR (Invitrogen).

Using the cDNA as template, relative *NOS* mRNA abundance was measured by qPCR using gene specific primers, Power SYBR Green PCR Master Mix (Applied Biosystems), and a Bio Rad CFX Connect Real-Time Detection System (Hercules, CA, USA). The ribosomal protein gene, *RpS7*, was used as the reference, and relative mRNA quantification was done using the 2^−∆∆CT^ method (Coggins, et al. 2012; Livak and Schmittgen 2001). The absence of genomic DNA in the cDNA was confirmed by melting curve analysis. The primers were as used in our earlier study: *Rps7* (AGAP010592): GACGGATCCCAGCTGATAAA and GTTCTCTGGGAATTCGAACG; *NOS* (AGAP029502): CAAGAGTGGGACCACATCAA and ACCCTTCTGGACCATCTCCT (Estevez-Lao, et al. 2020). Three independent trials originating from three different egg batches were conducted.

### NADPH diaphorase staining and imaging

Nitric oxide synthase (NOS) activity was detected using NADPH diaphorase staining as described (Estevez-Lao, et al. 2020). This reaction results in a purple precipitate in locations with NOS activity. Mosquitoes were injected 16% paraformaldehyde and the dorsal abdomens were collected in PBS containing 0.1% Tween 20. Abdomens were placed in 2 mL microcentrifuge tubes and washed with 50 mM Tris-buffered saline (TBS, pH 7.2) for 10 min. Abdomens were fixed again in 4% paraformaldehyde in TBS for 30 min, washed 3 times with TBS for 10 min each, and permeabilized in 0.2% Triton-X-100 in TBS for 15 min. Abdomens were then incubated for 90 min at 37 °C in 1 mM β-NADPH, 0.2 mM nitroblue tetrazolium, 0.2% Triton X-100 and 0.02% sodium azide in TBS, and washed 3 times in TBS for 10 min each. Each of the above steps was conducted in an orbital shaker. The dorsal abdomens were then mounted on glass slides with Vectashield Antifade Mounting Medium and Z-stacks were captured in 20 µm increments using bright field trans-illumination using the Nikon Eclipse Ni-E microscope and a Nikon Digital Sight DS-Fi1 digital color camera. For qualitative analysis of diaphorase staining, 8–10 abdomens were analyzed per treatment group.

### Analysis of mosquito heart physiology

Mosquitoes were anesthetized by placing them for < 60 s at −20 °C and then transferred to a Sylgard 184 silicone elastomer plate (Dow Corning Corp, Midland, MI). Each mosquito was restrained dorsal side up by placing 0.2 mm diameter Minutien Insect Pins at a 45° angle against (not through) each side of the anterior pronotal lobe of the thorax, and one pin through each wing (Andereck, et al. 2010; Glenn, et al. 2010). Mosquitoes were acclimated to room temperature and intravital videos of their dorsal abdomen were taken for 65 s under bright-field trans-illumination using a Nikon SMZ1500 stereo microscope, a Hamamatsu ORCA-Flash 2.8 digital CMOS camera (Hamamatsu Photonics, Hamamatsu, Japan), and Nikon Advanced Research NIS-Elements software. The first 60 s of each video was manually analyzed in NIS-elements to measure heart physiology. The following parameters were measured: (i) the total, anterograde and retrograde contraction rates; (ii) the percent contractions and percent time contracting in the anterograde and retrograde directions; and (iii) the frequency of heartbeat directional reversals (Andereck, et al. 2010; Estevez-Lao, et al. 2020; Glenn, et al. 2010). For each experiment, 5–6 independent trials were conducted. A total of 25–45 mosquitoes were analyzed per treatment group. Experiments were conducted on naïve (uninfected), injured (uninfected) and *E. coli*-infected mosquitoes because injury can have a small effect on the heart rate (Estevez-Lao, et al. 2020).

### Data availability and statistical analysis

All quantitative data are in Supplementary File 1. Statistical analyses were conducted using GraphPad Prism v 10.4.1 (GraphPad Software LLC, Boston, MA). Data on periostial hemocytes and heart physiology were analyzed by two-way ANOVA, followed by Tukey’s multiple comparisons test. Data on heart physiology are visually presented in three ways: (i) aggregated by infection status regardless of the type of liposome treatment, (ii) aggregated by the type of liposome treatment regardless of infection status, and (iii) unaggregated.

## Results

### Clodronate liposomes ablate sessile hemocytes without impacting pericardial cells or heart integrity

**Fig. 1:**
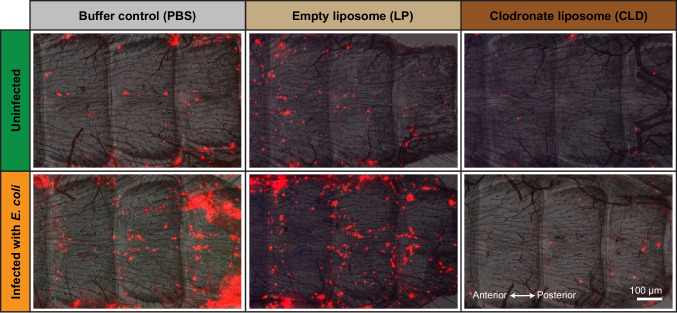
Clodronate liposomes ablate the sessile hemocytes. Fluorescence and bright field overlays showing several abdominal segments of mosquitoes that have been treated with PBS, LP, or CLD and then either left uninfected or have been infected with *E. coli* for 24 h. Hemocytes are labeled red with Vybrant CM-DiI

To determine whether clodronate liposomes affect sessile hemocytes, pericardial cells or the integrity of myocardial cells, we injected mosquitoes with PBS (buffer control), empty liposomes (LP; liposome control) or clodronate liposomes (CLD) and used fluorescence microscopy to visualize hemocytes, pericardial cells and the heart in the presence and absence of infection. Staining of hemocytes with CM-DiI demonstrated that CLD, but not LP or PBS, drastically reduces or eliminates the sessile hemocytes attached to the dorsal abdomen of uninfected and infected mosquitoes (Fig. 1). Because CLD was intrathoracically injected and disseminates throughout the hemocoel with the flow of hemolymph, from these data and published data showing CLD-mediated hemocyte depletion (Adegoke, et al. 2024; Cardoso-Jaime and Dimopoulos 2025; Kwon and Smith 2025), we infer that CLD ablates all hemocytes—circulating and sessile—throughout the entire body.

**Fig. 2:**
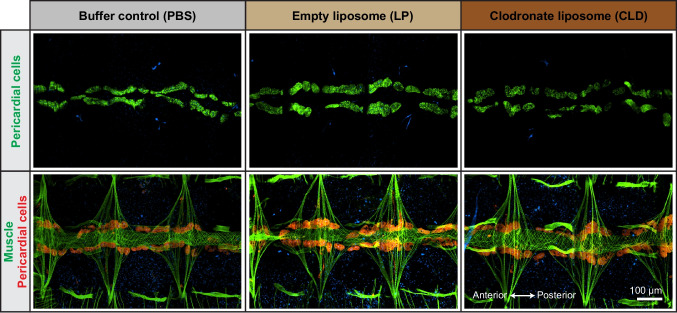
Clodronate liposomes do not affect the pericardial cells or the integrity of the heart. Fluorescence overlays showing several abdominal segments of mosquitoes that have been treated with PBS, LP, or CLD. In the top row, the pericardial cells have been labeled green with IgG-488. In the bottom row, the muscle has been labeled green with phalloidin-488 and the pericardial cells red with IgG-568. In both rows, nuclei are labeled blue with Hoechst 33342

Staining of pericardial cells by injecting fluorescently labeled IgG antibody demonstrated that neither CLD, LP or PBS affects the pericardial cells (Fig. 2). That is, the pericardial cells continue to flank the heart along its entire length. Finally, staining of muscle by injecting fluorescently labeled phalloidin demonstrated that the structure of the heart is unaffected by CLD, LP or PBS (Fig. 2). That is, the myocardial cells retain their spiral arrangement, and the heart remains attached to the tergum by alary muscles that span the junctions between abdominal segments.

### Clodronate liposomes ablate the periostial hemocytes

**Fig. 3:**
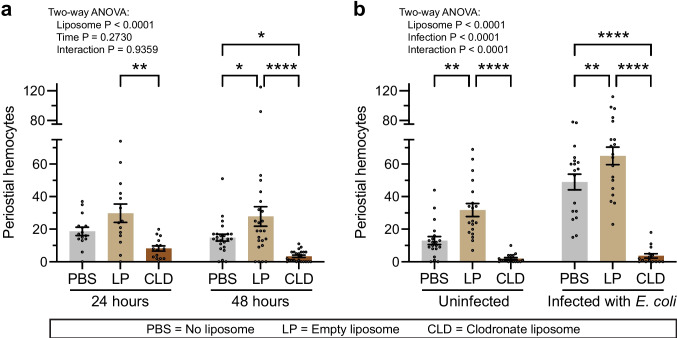
Clodronate liposomes ablate the periostial hemocytes. **a** Number of periostial hemocytes in mosquitoes treated with PBS, LP, or CLD for 24 or 48 h. **b** Number of periostial hemocytes in mosquitoes treated with PBS, LP, or CLD, left uninfected or infected with *E. coli* 24 h later, and hemocytes counted 24 after immune treatment. The graphs display the mean ± S.E.M., and circles denote the values of individual mosquitoes. Tukey’s multiple comparisons *P*: *P < 0.05, **P < 0.01, ****P < 0.0001

Because the predominant locations where sessile hemocytes aggregate are the periostial regions of the heart (King and Hillyer 2013), we quantified whether PBS, LP or CLD affects the number of periostial hemocytes in uninfected mosquitoes at 24 and 48 h after treatment (Fig. 3a). Relative to mosquitoes injected with PBS, the number of periostial hemocytes in LP-treated mosquitoes increased by 59% and 87% by 24 and 48 h after treatment, respectively. This indicates that liposomes by themselves are immunostimulatory and trigger periostial hemocyte aggregation. Relative to mosquitoes injected with LP, the number of periostial hemocytes in CLD-treated mosquitoes decreased by 73% and 88% by 24 and 48 h after treatment, respectively. Therefore, the clodronate in CLD kills the periostial hemocytes, and the effect lasts at least 48 h.

Because an infection, stimulates the aggregation of hemocytes within the periostial regions (King and Hillyer 2012), we next tested the effect of PBS, LPS, and CLD in the presence and absence of an infection (Fig. 3b). For this experiment, mosquitoes were treated with PBS, LP or CLD, and 24 h later, were left uninfected or were infected with *E. coli*. The number of periostial hemocytes was then counted 24 h after this latter treatment. The results in uninfected mosquitoes recapitulated the findings in our earlier experiment: relative to PBS, LP increased the number of periostial hemocytes by 145%, and relative to LP, CLD decreased the number of periostial hemocytes by 94%. We observed a similar result in infected mosquitoes: relative to PBS, LP increased the number of periostial hemocytes by 33%, and relative to LP, CLD decreased the number of periostial hemocytes by 95%.

The effect of liposome treatment was also notable when comparing uninfected and infected mosquitoes. In mosquitoes treated with PBS or LP, infection increased the number of periostial hemocytes by 276% or 104%, respectively. In contrast, CLD treatment ablated the periostial hemocytes in both uninfected and infected mosquitoes to a similar level, resulting in fewer than 4 periostial hemocytes per mosquito.

In summary, an infection induces periostial hemocyte aggregation, as has been previously demonstrated (King and Hillyer 2012, 2013; Meier, et al. 2024; Sigle and Hillyer 2016). These findings also demonstrate that, in uninfected and infected mosquitoes, empty liposomes are immunostimulatory and induce periostial hemocyte aggregation whereas clodronate liposomes destroy the periostial hemocytes.

### Hemocyte ablation alters immune responses on the surface of the heart

Periostial hemocytes destroy pathogens that circulate with the hemolymph (King and Hillyer 2012; Sigle and Hillyer 2016). Therefore, we examined whether hemocyte ablation alters immune patterns on the heart.

**Fig. 4:**
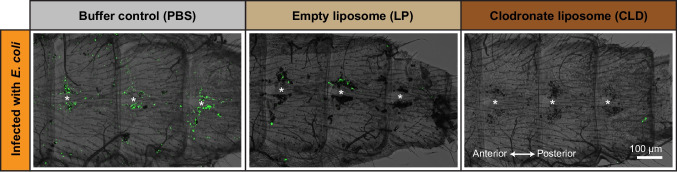
Hemocyte ablation modifies the localization of bacteria in the dorsal abdomen. Fluorescence and bright field overlays showing several abdominal segments of mosquitoes that have been treated with PBS, LP, or CLD and then infected with GFP-*E. coli* (green) for 24 h. Asterisks are placed on the lumen of the heart where the ostia (and periostial regions) are immediately above and below

We first examined the phagocytosis of live pathogens by visualizing the fluorescence of GFP-*E. coli* on the dorsal abdominal wall (Fig. 4). Aggregation of pathogens in the periostial regions is indicative of phagocytosis, as has been demonstrated using mosquitoes injected pHrodo-Red conjugated bacteria that only fluoresce after being phagocytosed (Sigle and Hillyer 2016). In PBS- and LP-treated mosquitoes, infection induced phagocytosis of *E. coli* by hemocytes, with the highest density of phagocytosis occurring in the periostial regions. In CLD-treated mosquitoes this pattern was disrupted: no noticeable pattern of *E. coli* accumulation was discernible, likely because no hemocytes remained in the periostial regions—or anywhere in the dorsal abdomen—to phagocytose *E. coli*. Instead, the bacteria continued to circulate with the hemolymph. Fluorescence was also not discernible in the abdomen of uninfected mosquitoes because there was no infection.

**Fig. 5:**
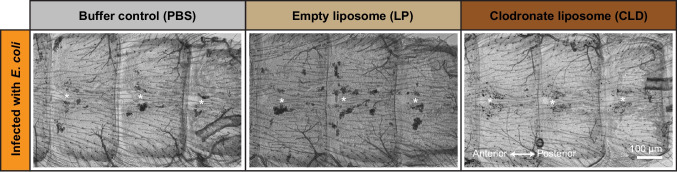
Hemocyte ablation modifies the localization of melanin in the dorsal abdomen. Bright field images showing several abdominal segments of mosquitoes that have been treated with PBS, LP, or CLD and then infected with *E. coli* for 24 h. Melanin is black. Asterisks are placed on the lumen of the heart where the ostia (and periostial regions) are immediately above and below

We next examined melanin accumulation in the dorsal abdomen (Fig. 5). In PBS and LP-treated mosquitoes, GFP-*E. coli* infection caused the aggregation of melanized pathogens on the dorsal abdominal wall, with most of the aggregation occurring within dense foci in the periostial regions. This is indicative of bacteria being melanized while in circulation and then phagocytosed by periostial hemocytes. In CLD-treated mosquitoes, melanin deposition still occurred around the heart, but the pattern was different. Whereas melanin accumulation in PBS- and LP-treated mosquitoes was densely packed at the periostial regions, melanin accumulation near the heart of CLD-treated mosquitoes was diffuse and more dispersed: some melanin was present within the periostial regions but also spanned laterally toward the pleuron. Based on this pattern, together with these mosquitoes not having periostial hemocytes (Fig. 3), melanized pathogens in CLD treated mosquitoes are not phagocytosed. Instead, melanized pathogens become stuck in the incomplete dorsal diaphragm created by the alary muscles, or small melanin particles are captured by pericardial cells (Fig. 2). Little or no melanin was observed in the abdomen of uninfected mosquitoes because no melanization immune response is taking place.

### Hemocyte ablation decreases NOS expression and NOS activity in the dorsal abdomen

During an infection, nitric oxide reduces the heart rate (Estevez-Lao, et al. 2020). Because nitric oxide synthase (NOS) catalyzes the conversion of L-arginine to produce L-citrulline and nitric oxide (Rivero 2006), we assessed whether liposome treatment alters *NOS* expression and NOS activity on the dorsal abdomen, which includes the heart.

**Fig. 6:**
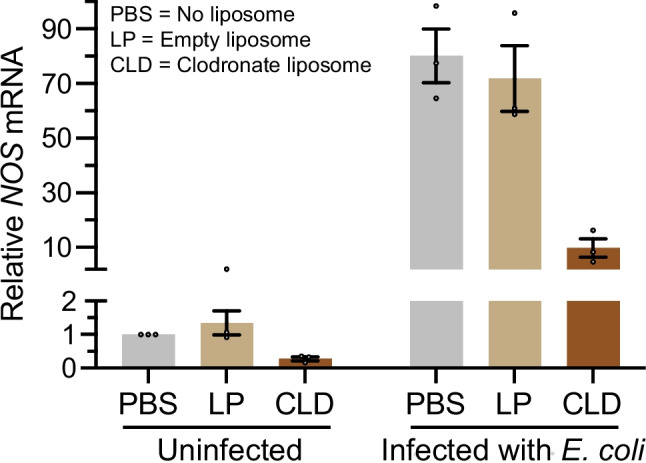
Hemocyte ablation reduces *NOS* mRNA abundance. *NOS* mRNA abundance measured by qPCR in mosquitoes treated with PBS, LP, or CLD in the absence of infection or 24 h after infection with *E. coli*. The graph displays the mean ± S.E.M. fold-change in mRNA level relative to naïve mosquitoes treated with PBS, using *Rps7* as the reference gene. Circles denote the values of individual biological trials

In uninfected mosquitoes, *NOS* mRNA abundance decreased when the hemocytes were ablated (Fig. 6). Relative to PBS, LP increased *NOS* mRNA abundance by 35%, which is consistent with the immunostimulatory activity of empty liposomes. Relative to LP, CLD decreased *NOS* mRNA abundance by 79%, which is consistent with the elimination of hemocytes known to produce *NOS* (Estevez-Lao, et al. 2025, 2020).

When a mosquito is infected, *NOS* mRNA abundance increased regardless of liposome treatment—80%, 72% or 10% following treatment with PBS, LP or CLD, respectively—but *NOS* mRNA abundance remained much lower in mosquitoes that had their hemocytes ablated with CLD (Fig. 6). When the comparison was made among the infected mosquitoes, relative to PBS, LP decreased *NOS* mRNA abundance by 10%, and relative to LP, CLD decreased *NOS* mRNA abundance by 86%. The relative amount of *NOS* mRNA in CLD-treated mosquitoes was the smallest, and the detected *NOS* expression is likely by pericardial cells or other non-hemocyte cells (Bergmann, et al. 2024). Altogether, these data demonstrate that ablating the hemocytes reduces the abundance of *NOS* mRNA in the dorsal abdomen.

**Fig. 7:**
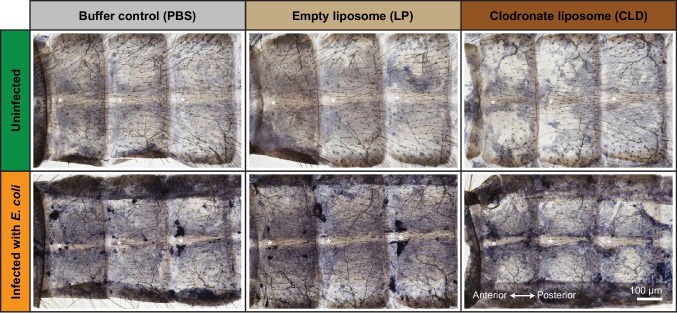
Hemocyte ablation eliminates NOS activity in the periostial regions. Bright field images showing several abdominal segments of mosquitoes that have been treated with PBS, LP, or CLD and then either left uninfected or have been infected with *E. coli* for 24 h. NOS activity was detected via diaphorase staining, which leaves a purple precipitate in locations with NOS activity. Asterisks are placed on the lumen of the heart where the ostia (and periostial regions) are immediately above and below

Having determined that hemocyte ablation reduces the abundance of *NOS* mRNA, we next visualized the localization of NOS activity in the dorsal abdomen via NADPH diaphorase staining (Fig. 7). In this procedure, the diaphorase enzymatic activity of NOS reduces tetrazolium dyes to a dark blue formazan precipitate in the presence of NADPH (Gonzalez-Zulueta et al. 2001). In uninfected mosquitoes, a low and diffuse level of NOS activity was detected throughout the abdomen, and there was no discernible difference between liposome treatments. In infected mosquitoes, NOS activity increased throughout the abdomen of mosquitoes treated with PBS, LP and CLD, which is consistent with the findings on mRNA abundance. In PBS- and LP- treated mosquitoes, intense foci of NOS activity were present in the periostial regions of the heart, which also coincided with areas of melanization. However, in CLD-treated mosquitoes, NOS activity was more broadly distributed, potentially due to hemocyte ablation enhancing more widespread NOS activity. Notably, intense NOS activity was absent on the heart of mosquitoes that had their hemocytes ablated with CLD. Therefore, ablating hemocytes using CLD reduces or eliminates the concentrated activity of NOS on the heart.

### Hemocyte ablation increases the heart rate and eliminates the infection-induced reduction in the heart rate

An infection reduces the heart rate via a nitric oxide-based mechanism (Estevez-Lao, et al. 2025, 2020). Because hemocytes produce nitric oxide (Estevez-Lao, et al. 2020; Hillyer and Estevez-Lao 2010), we tested whether treatment with PBS, LP or CLD alters the heart rate in uninfected and infected mosquitoes. We hypothesized that ablating periostial hemocytes increases the heart rate by decreasing NOS activity.

**Fig. 8:**
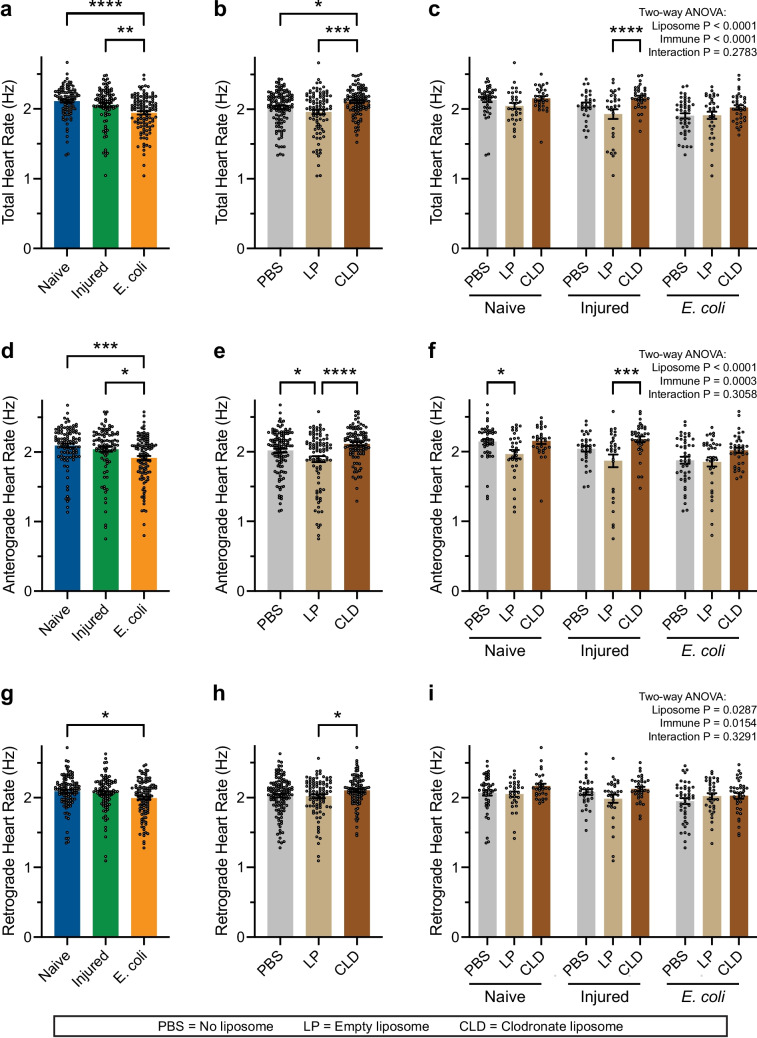
Hemocyte ablation increases the heart rate and eliminates the infection-induced reduction in the heart rate.** a**–**c** Total heart rate, with the data aggregated by immune status (**a**), liposome treatment (**b**), or unaggregated (**c**). **d**–**f** Anterograde heart rate, with the data aggregated by immune status (**d**), liposome treatment (**e**), or unaggregated (**f**). **g**–**i** Retrograde heart rate, with the data aggregated by immune status (**g**), liposome treatment (**h**), or unaggregated (**i**). The graphs display the mean ± S.E.M., and circles denote the values of individual mosquitoes. Tukey’s multiple comparisons *P*: *P < 0.05, **P < 0.01, ***P < 0.001, ****P < 0.0001

To begin the analysis, we first aggregated the data by infection status, regardless of the type of liposome, and confirmed that an infection decreases the heart rate: the heart of uninfected mosquitoes contracts at 2.11 Hz, which decreases 8% when a mosquito is infected (Fig. 8a). We then aggregated the data by liposome treatment, regardless of infection status, and discovered that LP decreases the heart rate whereas CLD increases it (Fig. 8b). The heart rate of PBS-treated mosquitoes contracts at 2.03 Hz. Relative to PBS-treated mosquitoes, the heart rate of LP-treated mosquitoes is 3% slower, and relative to LP-treated mosquitoes, the heart rate of CLD-treated mosquitoes is 8% faster. Finally, we disaggregated the data and uncovered that in naïve, injured and infected mosquitoes the heart rate was always highest in CLD treated mosquitoes (Fig. 8c).

The infection-induced reduction in the heart rate is more pronounced when contracting anterograde (Estevez-Lao, et al. 2020). Hence, we separated the anterograde and retrograde contraction periods and repeated the calculations (Fig. 8d–i). Aggregation of the data by infection status, regardless of the type of liposome, confirmed that an infection decreases the anterograde heart rate: the anterograde heart of uninfected mosquitoes is 2.09 Hz, and infection decreases it by 9%. Then aggregation of data by liposome treatment, regardless of infection status, revealed that, relative to PBS, LP decreases the anterograde heart rate by 6%, and relative to LP, CLD increases the anterograde heart rate by 12%. A similar but more attenuated finding was discovered for the retrograde heart rate. The retrograde heart rate in uninfected mosquitoes is 2.09 Hz, and an infection reduces the retrograde heart rate by 5%. Then aggregation of data by liposome treatment, regardless of infection status, revealed that, relative to PBS, LP decreases the retrograde heart rate by 1%, and relative to LP, CLD increases the heart rate by 4%. When we disaggregated the data for both the anterograde and retrograde heart rate, CLD-treated mosquitoes had the highest heart rate, regardless of whether the mosquito was naïve, injured or infected. Therefore, ablating hemocytes increases the heart rate and eliminates the infection-induced reduction in the heart rate. This cardiac phenotype is most pronounced when the heart is contracting anterograde.

### Hemocyte ablation does not alter the percentage of time the heart contracts anterograde versus retrograde or the frequency of heartbeat directional reversals

**Fig. 9:**
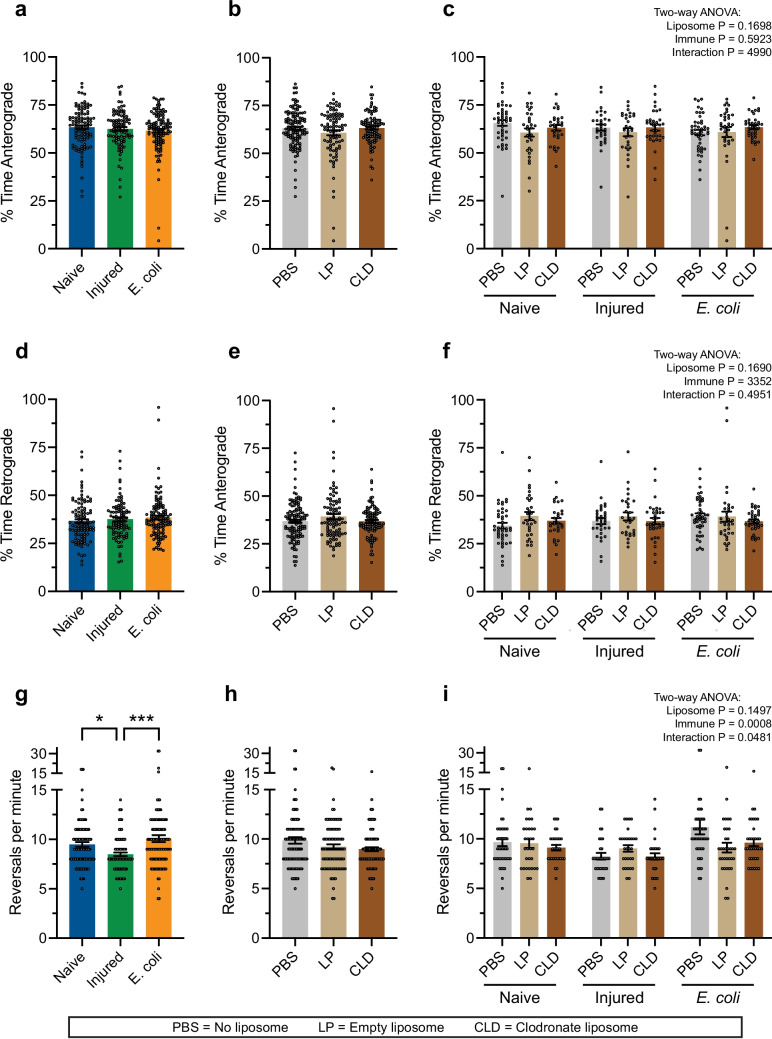
Hemocyte ablation does not modify the percentage of time the heart spends contracting anterograde versus retrograde or the frequency of heartbeat directional reversals. **a**–**c** Percent time the heart contracts anterograde, with the data aggregated by immune status (**a**), liposome treatment (**b**), or unaggregated (**c**). **d**–**f** Percent time the heart contracts retrograde, with the data aggregated by immune status (**d**), liposome treatment (**e**), or unaggregated (**f**). **g**–**i** Frequency of heartbeat directional reversals, with the data aggregated by immune status (**g**), liposome treatment (**h**), or unaggregated (**i**). The graphs display the mean ± S.E.M., and circles denote the values of individual mosquitoes. Tukey’s multiple comparisons *P*: *P < 0.05, ***P < 0.001

The heart of an adult mosquito periodically alternates between contracting anterograde and contracting retrograde (Andereck, et al. 2010; Estevez-Lao, et al. 2020; Glenn, et al. 2010). Hence, we calculated whether hemocyte ablation alters the proportion of time the heart spends contracting anterograde versus retrograde (Fig. 9a–f). The heart of naïve, injured and infected mosquitoes spent approximately the same amount of time contracting in the anterograde direction: 63%, 63% and 62%, respectively, with the remaining time spent contracting retrograde. Similarly, the heart of PBS-treated, LP-treated and CLD-treated mosquitoes spent 63%, 61% and 63% of the time contracting in the anterograde direction, respectively, with the remaining time spent contracting retrograde. Hence, neither the infection status nor the liposome treatment modifies the proportion of time spent contracting anterograde versus retrograde.

We then calculated whether hemocyte ablation alters the frequency of heartbeat directional reversals (Fig. 9g–i). We uncovered that an infection increases the frequency of heartbeat directional reversals, as we previously demonstrated (Estevez-Lao, et al. 2020): relative to naïve mosquitoes, an injury decreased the frequency of reversals by 11%, and relative to injured mosquitoes, an infection increased the frequency of reversals by 19%. Liposome treatment, however, did not meaningfully modify heartbeat directional reversals: relative to PBS, LP decreased the frequency of reversals by 6%, and relative to LP, CLD decreased the frequency of reversals by 2%.

In summary, infection does not alter the proportion of time spent contracting anterograde versus retrograde but decreases the frequency in which the heart switches from contracting anterograde to contracting retrograde and vice versa. Hemocyte ablation does not alter the proportion of time spent contracting anterograde versus retrograde or the frequency of heartbeat directional reversals.

## Discussion

**Fig. 10:**
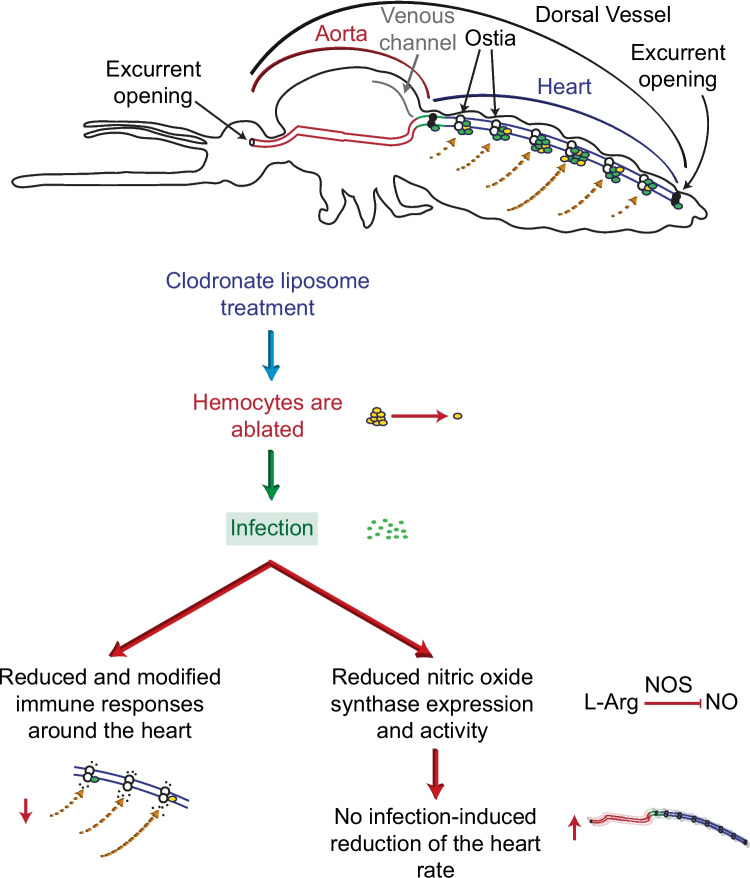
Diagrammatic representation of the effect of hemocyte ablation on immune responses and the heart rate

Periostial hemocytes reside on the surface of the heart, where they survey the hemolymph for invading pathogens (King and Hillyer 2012; Sigle and Hillyer 2016). Here, we demonstrate that clodronate liposomes destroy the sessile hemocytes—including the periostial hemocytes that associate with the heart—while leaving pericardial and myocardial cells unaffected. Moreover, we conclusively demonstrate that periostial hemocytes drive immune responses on the heart and modulate circulatory physiology via a nitric oxide-dependent mechanism (Fig. 10).

Clodronate liposomes have been used to deplete hemocytes in mosquitoes, fruit flies, moths, ticks and snails, resulting in animals that are immunocompromised (Adegoke, et al. 2023; Bergamini, et al. 2023; Dijokaite, et al. 2021; Kwon and Smith 2019). Clodronate liposomes are ingested by phagocytes, the liposomes are digested, and the clodronate that is released kills the cell by inducing apoptosis (van Rooijen and Hendrikx 2010). Clodronate eliminates both circulating and sessile hemocytes (Adegoke, et al. 2024; Cardoso-Jaime and Dimopoulos 2025; Kwon and Smith 2025). Here, we show that the apoptosis of sessile hemocytes is complete by 24 h of CLD administration. Having so many apoptotic bodies produced at one time could affect other tissues, but the timeline of their clearance is unknown. Given that hemocytes begin phagocytosing bacteria within 5 min of their introduction into the hemolymph (Hillyer, et al. 2003a, 2003b), we speculate that hemocyte death occurs quickly after CLD introduction, and that apoptotic body clearance is completed well ahead of the 24 h timepoint used in our experiments. But hemocytes are not the only immunocompetent cells in mosquitoes. Pericardial cells filter the hemolymph and produce immune factors (Bergmann, et al. 2024; Cardoso-Jaime, et al. 2021, 2023; Hernandez-Martinez, et al. 2013; King and Hillyer 2012). Our experiments demonstrate that clodronate ablates the sessile hemocytes—including the periostial hemocytes—while leaving the pericardial cells and other heart-associated cells unaffected.

During an infection, periostial hemocytes phagocytose pathogens in areas of high hemolymph flow (Sigle and Hillyer 2016). This includes live pathogens as well as pathogens that have been melanized (King and Hillyer 2012; League and Hillyer 2016; Sigle and Hillyer 2016). Here, we demonstrate that hemocyte ablation eliminates the phagocytosis of bacteria within the periostial regions. This provides another layer of evidence that periostial hemocytes, and not pericardial cells, are the major phagocytes on the heart. Moreover, although melanin has been detected inside pericardial cells (Hernandez-Martinez, et al. 2013), we observed that melanin in CLD-treated mosquitoes was more diffuse and also spanned laterally away from the heart and the pericardial cells. This is unlike the dense foci of melanin that form within the periostial regions of mosquitoes that have hemocytes. We propose that in hemocyte-depleted mosquitoes, non-melanized bacteria continue to circulate with the hemolymph but melanized bacteria, which are larger, become entrapped in the incomplete dorsal diaphragm that is formed by the alary muscles that tether the heart to the dorsal abdominal wall.

Circulating and sessile hemocytes—including the periostial hemocytes—produce nitric oxide (Bergmann, et al. 2024; Estevez-Lao, et al. 2020; Hillyer and Estevez-Lao 2010). But other cells in the body also produce this free radical. For example, midgut cells produce nitric oxide to combat a malarial infection, and pericardial cells do so in response to a bacterial infection (Bergmann, et al. 2024; Luckhart, et al. 1998). Nitric oxide is produced by NOS (Rivero 2006), and we uncovered that *NOS* expression and NOS activity on the dorsal abdominal wall is markedly reduced when the hemocytes are ablated. Hemocytes are a small proportion of the cells on the dorsal abdominal wall, so we infer that hemocytes are the primary producers of NOS on the dorsal abdominal wall. Particularly notable is that hemocyte ablation eliminates NOS activity in the periostial regions, indicating that hemocytes are primary producers of nitric oxide on the surface of the heart. But an infection increases *NOS* expression and NOS activity throughout the abdomen, even when hemocytes are ablated. In mosquitoes without hemocytes, we predict that other cells produce NOS and other immune effectors in greater quantities than normal to compensate for the lack of hemocyte-mediated immune responses.

An infection induces the migration of hemocytes to the heart, and this is driven by the IMD and JNK pathways (Yan, et al. 2022). Heart associated hemocytes produce nitric oxide, and inhibiting nitric oxide production using the chemical inhibitor L-NAME reduces the ability of mosquitoes to control a systemic bacterial infection and prevents the infection induced reduction in the heart rate (Estevez-Lao, et al. 2020; Hillyer and Estevez-Lao 2010). Moreover, in uninfected mosquitoes, activation of the IMD pathway increases NOS activity on the heart and decreases the heart rate (Estevez-Lao, et al. 2025). Together, these studies tie an increase in the number of periostial hemocytes to an increase in nitric oxide production and cardiac deceleration. A nitric oxide-based decrease in the heart rate has also been observed in fruit flies and stick insects (Broderick, et al. 2006; da Silva, et al. 2012). In locusts an opposite phenotype occurs: hemocytes on the heart produce nitric oxide, but this causes an increase in the heart rate (Bergmann, et al. 2021; Bullerjahn, et al. 2006). Here, we uncovered that ablating the hemocytes, which reduces *NOS* expression and NOS activity on the heart, increases the heart rate and eliminates the infection induced reduction in the heart rate, with this being particularly accentuated when the heart contracts anterograde. This directional effect is notable because a similar predilection occurs after infection (Estevez-Lao, et al. 2020). The phenotype can be explained by how cardiac structures are differentially used between anterograde and retrograde contracting periods: periostial hemocytes reside around abdominal ostia that are only open when the heart contracts anterograde, whereas the thoraco-abdominal ostia—which are largely devoid of hemocytes—are open when the heart contracts retrograde (Glenn, et al. 2010; Sigle and Hillyer 2018a). If hemocytes are making nitric oxide that modulates heart physiology, then it stands to reason that the effect is greater when the hemolymph is flowing toward the heart, which occurs during anterograde contraction periods. The physiological benefit of having a lower heart rate when a mosquito is infected has not been conclusively deciphered, but an intriguing possibility is that slowing the flow of hemolymph increases the time that periostial hemocytes have to identify, capture and phagocytose pathogens as they approach the heart.

Lysozymes can also reduce the heart rate, and this may be via a NOS-mediated mechanism (Estevez-Lao, et al. 2020). In canines, lysozymes can cause cardiovascular dysfunction via a hydrogen peroxide-dependent pathway that appears to activate NOS (Mink, et al. 2009). On the heart of mosquitoes, pericardial cells also produce lysozymes and nitric oxide (Bergmann, et al. 2024; Cardoso-Jaime, et al. 2021). Because the infection-induced cardiac phenotypes are only observed when hemocytes are present, lysozymes or nitric oxide produced by pericardial cells do not meaningfully alter the circulatory physiology of mosquitoes.

In conclusion, the periostial hemocytes, and not the pericardial cells, are the primary drivers of immune responses on the heart of mosquitoes. Moreover, nitric oxide produced by periostial hemocytes, and not pericardial cells, reduces the heart rate. 

## Supplementary Information

Below is the link to the electronic supplementary material.

ESM 1 (XLSX 34.5 KB)

## Data Availability

All quantitative data generated in this study are presented in Supplementary file 1.
